# Letter to the Editor: Silicon Reference Materials Certified for Isotope Abundances

**DOI:** 10.6028/jres.096.038

**Published:** 1991

**Authors:** S. Valkiers, P. De Bièvre, G. Lenaers, H. S. Peiser

**Affiliations:** Central Bureau for Nuclear Measurements, Commission of the European Communities, Joint Research Centre, B-2440, Geel, Belgium; University of Antwerpen, Chemistry Department, B-2610 Antwerpen-Wilrijk, Belgium; National Institute of Standards and Technology, Gaithersburg, MD 20899

**Keywords:** absolute abundances, atomic weights, isotope abundances, isotopic mass spectrometry, isotope reference materials, silicon, silicon tetrafluoride

## Abstract

In a series of gas mass-spectrometric measurements performed near the highest attainable accuracy, samples from two highly homogeneous batches of silicon crystals and silica powder were compared directly with a synthetic mixture of the three stable isotopes of silicon. Thereby, this work not only established the “absolute” atomic weight of these batches, but also makes portions of these batches available as an Isotopie Reference Material for accurate isotopic abundance measurements in geochemical and other isotope-abundance studies of silicon.

A precise determination of differences of a property between similar specimens is more easily achieved than absolute accuracy in the measurement. In mass-spectrometric determinations of isotope abundances, variations are often small—but significant relative to achievable precision—between specimens from differing chemical, physical, geological, or biological processes. It is just these variations that open new fields of study in geochemistry, chemical technology, solid-state physics, archeology, analysis of trace elements, etc. Thus, as in other fields, credible isotope reference materials (IRMs) are needed which must fulfill the following criteria:

- near perfect homogeneity within and between samples of the IRM;
- wide and open availability of IRM samples in adequate amounts so that different laboratories can refer to the “same” material;
- full chemical and physical characterization of the IRM; and
- direct absolute measurement of the isotopic composition of the IRM near the highest contemporary accuracy.

The fourth criterion demands a direct atomic-weight and isotopic composition determination recognized to have been carried out near the highest accuracy so far achieved. That in turn implies the preparation of synthetic mixtures from highly enriched isotope constituents. During the past 25 years such measurements have been rarely made and only on a total of about 15 elements. The introduction therefore of two new silicon IRMs is an event of note.

A smaller batch of small silicon single crystals (total mass ~10 g) and a larger batch of SiO_2_ grains (10 kg Optipur Merck^1^) have been characterized for their isotopic homogeneity, isotopic composition and relative atomic weight, *A*_r_(Si). This has resulted in two IRMs: IRM-017 (Si) and IRM-018 (SiO_2_), portions of which are now available from the Central Bureau for Nuclear Measurements (CBNM).

The isotopie measurements on the three stable isotopes of silicon were performed by gas isotope mass spectrometry [1] on samples which were converted to SiF4 gas by the following chemical steps carried out on a large number of portions. In step 1 samples of the silicon candidate reference material were dissolved in warm dilute sodium hydroxide, and the solution of sodium silicate was converted to aqueous silicic acid in an acidic ion exchanger. Water from that solution was evaporated and the residue heated to 950 °C to form silica (SiO_2_). Steps 2 and 3 were applied to samples of both candidate reference materials. In step 2 the silica was dissolved in hydrofluoric acid from which barium hexafluosilicate was precipitated. In step 3 the barium salt was heated to 540 °C in an evacuated vessel, and the silicon tetrafluoride was frozen out.

The isotope-abundance ratio measurements on SiF_4_ (^29^SiF_3_^+^/^28^SiF_3_^+^,^30^SiF_3_^+^/^28^SiF_3_^+^) were calibrated by means of a gravimetric mixture of the three enriched isotopes [2]. The amounts of the enriched isotopes used to prepare the mixture were chosen such that the prepared ratios were close to those for natural silicon. With this synthetic isotope mixture the mass-spectrometric correction factors *K*(*K* = ratio as prepared/ratio as observed) for the observed ratios were determined. The IRMs were measured in the same series of tightly controlled measurements from which the effect of any inevitable instrumental drift could be minimized. In this way the above correction factors could be applied to these IRM measurements, resulting in “absolute” isotope abundances and atomic weights for both materials (Table 1). The measurement technique for gas isotopie measurements as it was developed by the authors for this work will be described in more detail in other future publications. Similarly, the required chemical preparation techniques of the samples deserve fuller description and critical analysis elsewhere. The atomic-weight measurements here described are in experimental agreement with the previously recognized best silicon measurements by Barnes et al. [4]. It was possible to include a sample from those earlier measurements in the self-same series described above. The consistency of all these measurements was thereby further established.

## Figures and Tables

**Table 1:** Isotopic Composition and *A*_r_(Si) for both IRMs. Uncertainties are indicated under the digits to which they relate and are computed on a two-standard deviation basis. (The mass spectrometric measurements on isotopic reference materials 017 and 018 were carried out during a period just before a filament change became necessary. An increasing instability of the old filament was mainly responsible for the larger uncertainties when compared with those for an entirely different silicon crystal that happens to have a virtually identical relative atomic weight.)

	CBNM-IRM-017 Si	CBNM-IRM-018 SiO_2_
Molar isotope abundance ratios		
^29^Si/^28^Si	0.050 69	0.050 83
	12	12
^30^Si/^28^Si	0.033 52	0.033 60
	10	10

Molar abundances (fractional)		
^28^Si	0.922 33	0.922 14
	14	14
^29^Si	0.046 75	0.046 88
	11	11
^30^Si	0.030 92	0.030 98
	8	8

Mass percentages		
^28^Si	91.877	91.857
	14	14
^29^Si	4.823	4.836
	11	11
^30^Si	3.300	3.307
	8	8

Relative atomic mass (atomic weight)		
*A*_r_(Si)	28.085 40	28.085 65
	19	19

## Footnotes

1 Certain commercial equipment, instruments, or materials are identified in this paper to specify adequately the experimental procedure. Such identification does not imply recommendation or endorsement by the National Institute of Standards and Technology, nor does it imply that the materials or equipment identified are necessarily the best available for the purpose.
